# High-pitch, 120 kVp/30 mAs, low-dose dual-source chest CT with iterative reconstruction: Prospective evaluation of radiation dose reduction and image quality compared with those of standard-pitch low-dose chest CT in healthy adult volunteers

**DOI:** 10.1371/journal.pone.0211097

**Published:** 2019-01-24

**Authors:** Hyun Kyung Lim, Hong Il Ha, Hye Jeon Hwang, Kwanseop Lee

**Affiliations:** 1 Department of Radiology, Soonchunhyang University Hospital Seoul, Seoul, Republic of Korea; 2 Department of Radiology, Hallym University Medical Center, Hallym University Sacred Heart Hospital, Anyang-si, Gyeonggi-do, Republic of Korea; University of Oklahoma, UNITED STATES

## Abstract

**Purpose:**

Objective of this study was to evaluate the effectiveness of the iterative reconstruction of high-pitch dual-source chest CT (IR-HP-CT) scanned with low radiation exposure compared with low dose chest CT (LDCT).

**Materials and methods:**

This study was approved by the institutional review board. Thirty healthy adult volunteers (mean age 44 years) were enrolled in this study. All volunteers underwent both IR-HP-CT and LDCT. IR-HP-CT was scanned with 120 kVp tube voltage, 30 mAs tube current and pitch 3.2 and reconstructed with sinogram affirmed iterative reconstruction. LDCT was scanned with 120 kVp tube voltage, 40 mAs tube current and pitch 0.8 and reconstructed with B50 filtered back projection. Image noise, and signal to noise ratio (SNR) of the infraspinatus muscle, subcutaneous fat and lung parenchyma were calculated. Cardiac motion artifact, overall image quality and artifacts was rated by two blinded readers using 4-point scale. The dose-length product (DLP) (mGy∙cm) were obtained from each CT dosimetry table. Scan length was calculated from the DLP results. The DLP parameter was a metric of radiation output, not of patient dose. Size-specific dose estimation (SSDE, mGy) was calculated using the sum of the anteroposterior and lateral dimensions and effective radiation dose (ED, mSv) were calculated using CT dosimetry index.

**Results:**

Approximately, mean 40% of SSDE (2.1 ± 0.2 mGy vs. 3.5 ± 0.3 mGy) and 34% of ED (1.0 ± 0.1 mSv vs. 1.5 ± 0.1 mSv) was reduced in IR-HP-CT compared to LDCT (*P* < 0.0001). Image noise was reduced in the IR-HP-CT (16.8 ± 2.8 vs. 19.8 ± 3.4, P = 0.0001). SNR of lung and aorta of IR-HP-CT showed better results compared with that of LDCT (22.2 ± 5.9 vs. 33.0 ± 7.8, 1.9 ± 0.4 vs 1.1 ± 0.3, *P* < 0.0001). The score of cardiac pulsation artifacts were significantly reduced on IR-HP-CT (3.8 ± 0.4, 95% confidence interval, 3.7‒4.0) compared with LDCT (1.6 ± 0.6, 95% confidence interval, 1.3‒1.8) (*P* < 0.0001). SNR of muscle and fat, beam hardening artifact and overall subjective image quality of the mediastinum, lung and chest wall were comparable on both scans (*P* ≥ 0.05).

**Conclusion:**

IR-HP-CT with 120 kVp and 30 mAs tube setting in addition to an iterative reconstruction reduced cardiac motion artifact and radiation exposure while representing similar image quality compared with LDCT.

## Introduction

Low-dose chest computed tomography (LDCT) is widely used to screen for lung cancer; the utility thereof became clear during the National Lung Screening Trial [1]. However, repeated CT scans are inevitably associated with increased radiation doses, although the mean radiation dose per scan is only approximately 1.5 mSv [2, 3]. As low-level radiation-related carcinogenesis is stochastic in nature, the radiation exposure associated with screening CT has become of concern, despite the utility of CT for lung cancer evaluation [2, 4]. Lung tissue status is assessable by CT using reduced radiation doses; the consistency of air within the lungs and the pulmonary parenchyma differ significantly. Many studies seeking to reduce the LDCT radiation dose even further are available [5–8]. The approaches include reductions in tube current and voltage, automatic tube current modulation, and the use of new hardware such as selective in-plane shielding [9–11]. Current efforts are focused principally on the development of image reconstruction algorithms. Iterative reconstruction (IR) has been employed to reduce LDCT image noise, the incidence of artifacts, and false-negative findings, especially in low-current settings. In addition, IR allows for substantial radiation dose savings and improves image quality [5, 7, 8, 12, 13].

After the introduction of dual-source CT (DSCT), several studies used high-pitch scanning to reduce the radiation dose [14–16]. A major advantage of DSCT is able to increase pitch factor up to 3.2 results in improvement of temporal resolution during CT scan [17]. DSCT has two measurement systems consisting two x-ray tubes and corresponding detectors. Single source CT has only one x-ray tube and corresponding detector, thus gapless z-sampling is limited to a maximum spiral pitch value of 1.5. Image gap would occur at spiral pitch values larger than 1.5. However, second measurement system of DSCT provides volume coverage without gaps at much higher pitch values. Thus, DSCT allows gapless sampling for a pitch value of 3.2 or less without image distortions inside the scan field of view (FOV) of second detector (34cm) [18]. The clinical benefits provided by DSCT have been exploited primarily for cardiac imaging [19–22]. Few studies have reported the effect of high-pitch DSCT on the image quality of the pulmonary parenchyma as well as reduction of cardiac motion when evaluating lung parenchyma [14, 15, 23]. The effects of iterative reconstruction of high-pitch dual-source chest CT (IR-HP-CT) and a reduced radiation dose on features of LDCT images other than cardiac pulsation artifacts have not yet been explored. Thus, the purpose of the present study was to evaluate the utility of 120 kVp/30 mAs IR-HP-CT, on radiation dose reduction, suppression of cardiac pulsation, and image quality, compared with standard-pitch LDCT in healthy adult volunteers.

## Materials and methods

### Patients

This prospective case-control study complied with the Declaration of Helsinki and was approved by the institutional review board and ethics committee of the Hallym University Sacred Heart Hospital; Written informed consent was obtained from all volunteers. From February to April 2015, 30 healthy adult volunteers (23 males and 7 females; mean age 44.0 years; range 34‒71 years) were enrolled. The mean body mass index was 24.0 kg/m^2^ (range, 18.1‒28.2 kg/m^2^). The volunteers were not charged for CT or medical consultation, but they received no other benefit (financial or otherwise). The total effective radiation exposure did not exceed 3 mSv.

### CT scan protocols

All CT scans were performed using a second-generation DSCT scanner (Somatom Definition FLASH; Siemens Healthcare, Forchheim, Germany). The scan protocols are summarized in Table 1. The pitch and gantry rotation time were determined by the software provided by the manufacturer. The 3.2 pitch is the highest value enable on the machine. If the pitch is set to 3.2, the gantry rotation is automatically fixed to 0.28 seconds. The tube voltage was fixed at 120 kVp on both scans. The automatic tube current modulation algorithm was not applied to evaluate the fixed mAs effect. Because high-pitch DSCT with tube current of 40mAs and filtered back projection had been proved to be effective in previous study, tube current of 30 mAs combined with IR was applied in this study to reduce the radiation dose based on literature review [23]. Effective tube current of LDCT was fixed at 40 mAs. All scans were craniocaudal in direction, and the lengths of the two scans from each volunteer were identical using same FOV on CT console. Each scan was performed at a collimation of 64 × 0.6 mm and with slice acquisition dimensions of 128 × 0.6 mm (using a z-flying focal spot). No intravenous contrast material was injected

**Table 1:** CT scan parameters for LDCT and IR-HP-CT.

	LDCT	IR-HP-CT
Pitch	0.8	3.2
Gantry rotation time (ms)	0.5	0.28
Reconstruction algorithm (kernel)	Filtered back projection (B50f)	Iterative reconstruction (I50f, SAFIRE, S3)
Tube current (mAs)	40	30
Tube voltage (kVp)	Fixed 120 kVp	
Automatic tube current modulation	Not applied	

LDCT, low-dose chest CT; IR-HP-CT, iterative reconstruction of high-pitch dual-source chest CT; SAFIRE, Sinogram Affirmed Iterative Reconstruction.

### Image reconstruction

The IR-HP-CT images were reconstructed using an IR algorithm (Sinogram-affirmed iterative reconstruction [SAFIRE], strength S3; Siemens Healthcare); the I50f iterative kernel was employed to evaluate the lung and mediastinum. LDCT images were reconstructed using filtered back projection algorithm (kernel B50f). Both IR-HP-CT and LDCT raw image data were reconstructed with a slice thickness of 3mm and an interval of 3mm. The consecutive CT examinations were dicomized and sent to our Picture Archiving and Communication System (PACS; Infinitt Healthcare, Seoul, Korea). All images sets were displayed on the default pre-selected lung window setting (window width 1500 HU, window level−700 HU) and mediastinum setting (window width 350 HU, window level 35 HU) that did not allow any change.

### Radiation dose analysis

The dose-length product (DLP) (mGy∙cm) were obtained from each CT dosimetry table. Scan length was calculated from the DLP results. The DLP parameter was a metric of radiation output, not of patient dose. Size-specific dose estimation (SSDE) was calculated using the sum of the anteroposterior and lateral dimensions according to the American Association of Physicists in Medicine (AAPM) released report 204 [24].

### Image quality analysis

#### Objective analysis

Image noise was objectively measured by calculating the standard deviation of CT attenuation in regions of interest (ROIs) drawn on the PACS; the ROIs were in the ascending aorta at the level of the carina, subcutaneous fat of the anterior chest wall, and the left infraspinatus muscle at the level of the azygos vein. The signal-to-noise ratio (SNR) of these regions and the lung parenchyma were calculated. We ensured that the ROIs were identical between paired CT images.

#### Cardiac pulsation artifacts

Cardiac pulsation artifacts were assessed by a single board-certified radiologist using a four-point scale. The pulmonary vessels, bronchial wall, mediastinal pleural line of the lung and blurring of cardiac margin were evaluated as follows: 4, sharp with no blurring; 3, minimal blurring and borderline pulsation artifacts <3 mm in length; 2, blurring of any bronchus or pulmonary vessel ≥3 mm but <5 mm in length caused by borderline pulsation artifacts; and 1, blurring of any bronchus or pulmonary vessel or borderline pulsation artifacts ≥5 mm in length [23]. A score of >2 is considered as acceptable for diagnosis.

#### Subjective analysis

All CT images were randomized and evaluated independently by two board-certified radiologists (with 10 and 6 years of experience) blinded to the patient information and image parameters. The features evaluated in the subjective image quality analysis are those of the modified European guideline [25] and are summarized in Table 2. The overall image quality of the lung, mediastinum, and chest wall and the sharpness of reproduction of mediastinal structures and the chest wall were scored on a four-point scale as follows: 1, poor; 2, suboptimal; 3, adequate; 4, excellent. Artifacts caused by photon deficiency (windmill, streak, and beam-hardening artifacts) were also ranked on a four-point scale as follows: 4, no or minimal artifacts; 3, artifacts affecting a part of the segment without compromising the identification of boundaries between anatomical structures; 2, artifacts affecting the entire segment without compromising the identification of boundaries between anatomical structures; and 1, artifacts compromising the identification of boundaries between anatomical structures.

**Table 2:** Image quality features assessed.

Mediastinum	Overall image quality Sharp reproduction of the major mediastinal structures (e.g., the trachea, esophagus, superior vena cava, heart, aorta, and pulmonary artery)
Chest wall	Overall image quality Sharp reproduction of chest wall structures (e.g., the margins of the muscles and the fascia) Artifacts in the shoulder and chest wall
Lung	Overall diagnostic image quality of the parenchyma Cardiac pulsation artifacts Artifacts in the lung parenchyma Peripheral lung image sharpness

Visual assessment of pulmonary lesions involved evaluation of ground-glass opacity per se, emphysema or bullae, solid nodules, and nodules exhibiting ground-glass opacity. The presence or absence of pulmonary lesions and their locations, if present, were noted. Any disagreement was resolved by consensus.

### Statistical analysis

All statistical analyses were performed using MedCalc (ver. 16.8, MedCalc Software, Ostend, Belgium). The paired t-test or Wilcoxon signed-rank test was used to compare the objective image noise, SNR, radiation dose, cardiac pulsation artifacts, subjective image quality scores, and effectiveness of pulmonary lesion detection between IR-HP-CT and LDCT. The weighted kappa value with linear weights was used to assess the inter-rater agreement of the subjective image quality analysis. The significance level for all tests was 5% (two-sided).

## Results

### Radiation dose

**Fig 1:**
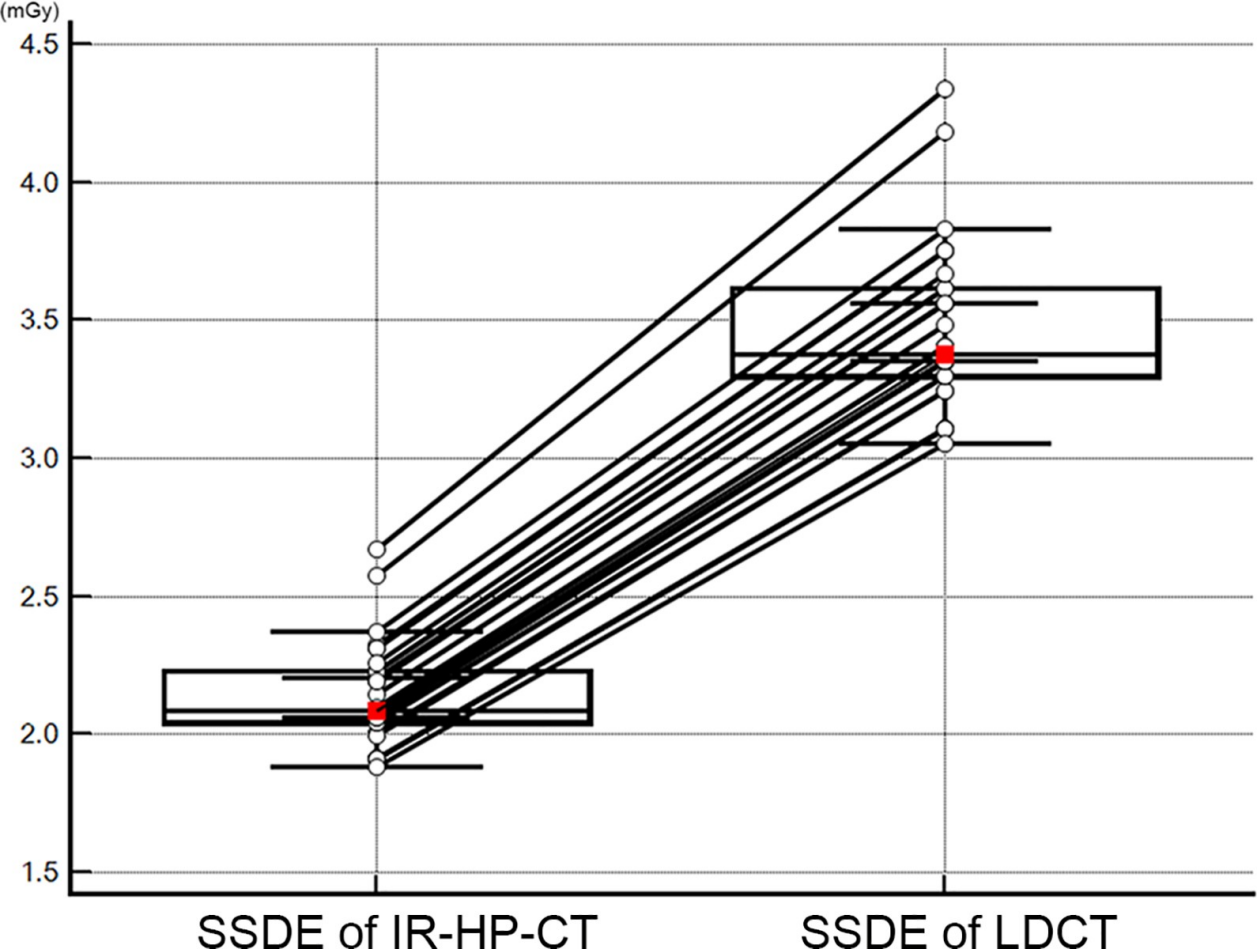
Size-specific dose estimates in each volunteer using LDCT and IR-HP-CT. The mean SSDE was significantly lower using IR-HP-CT (*P* < 0.001). The central box represents the values from the lower to upper quartile (25 to 75 percentile). The red squares are the means and the middle line represents the median.

The mean and standard deviation of anteroposterior and lateral diameter summation of volunteer, scan length, DLP, ED, and SSDE were summarized at Table 3. The mean scan length of IR-HP-CT was significantly longer than LDCT (*P* < 0.0001). The mean DLPs were 109.5 ± 7.3 mGy·cm (95% confidence interval (CI), 106.8‒112.2 mGy·cm) for LDCT and 73.1 ± 4.6 mGy·cm (95% CI, 71.4‒74.8 mGy·cm) for IR-HP-CT; the mean estimated ED were 1.5 ± 0.1 mSv (95% CI, 1.5‒1.6 mSv) and 1.0 ± 0.1 mSv (95% CI, 1.0‒1.1 mSv), respectively. The mean length of the anteroposterior and lateral diameter summation was 58.5 cm. The mean SSDE were 2.1 ± 0.2 mGy (95% CI, 2.1–2.2 mGy) for IR-HP-CT and 3.5 ± 0.3 mGy (95% CI, 3.4–3.6 mGy) for LDCT, thus approximately 40% reduction was achieved in the IR-HP-CT compared with that of LDCT (*P* < 0.0001) (Fig 1).

**Table 3:** Results of radiation dose.

	IR-HP-CT	LDCT	*P-*value*
Scan length (cm)	44.2 ± 2.8 (43.2, 45.3)	40.8 ±2.7 (39.8, 41.9)	< 0.0001
DLP (mGy·cm)	73.1 ± 4.6 (71.4, 74.8)	109.5 ± 7.3 (106.8, 112.2)	< 0.0001
SSDE (mGy)	2.1 ± 0.2 (2.1, 2.2)	3.5 ± 0.3 (3.4, 2.6)	< 0.0001
Effective radiation dose (mSv)	1.0 ± 0.1 (1.00, 1.05)	1.5 ± 0.1 (1.49, 1.57)	< 0.0001
Summation of anteroposterior and lateral diameter (cm)	58.5 ± 4.5 (57.0, 60.5)		

Data are means ± standard deviation, and the values in parentheses are the 95% confidence intervals. IR-HP-CT, iterative reconstruction of high-pitch dual-source chest CT; LDCT, low-dose chest CT; DLP, dose-length product; SSDE, size-specific dose estimates

* *P-*values were calculated using the paired t-test.

### Objective image quality

The mean quantitative image noise and SNR scores are summarized in Table 4. The image noise measured in three areas was significantly lower on IR-HP-CT than LDCT images (*P* < 0.001). The image noise was higher in peripheral areas (subcutaneous fat or the infraspinatus muscle) than in the central area (the ascending aorta) on both LDCT and IR-HP-CT images. The SNRs of all measured areas were significantly better on IR-HP-CT images (*P* < 0.0001).

**Table 4:** Results of objective image analysis using LDCT and IR-HP-CT.

	LDCT	IR-HP-CT	*P*-value*
Image noise			
Ascending aorta	41.1 ± 7.6 (38.3, 43.9)	25.7 ± 4.7 (23.9, 27.4)	< 0.0001
Subcutaneous fat	38.7 ± 7.8 (35.8, 41.6)	32.0 ± 5.7 (29.9, 34.2)	0.0003
Infraspinatus muscle	59.0 ± 13.2 (54.1, 63.9)	41.2 ± 6.0 (39.0, 43.4)	< 0.0001
Signal-to-noise ratio			
Ascending aorta	1.07 ± 0.26 (0.97, 1.17)	1.87 ± 0.37 (1.73, 2.01)	< 0.0001
Subcutaneous fat	3.18 ± 0.67 (2.92, 3.43)	5.05 ± 0.88 (4.72, 5.38)	< 0.0001
Infraspinatus muscle	0.98 ± 0.27 (0.88, 1.08)	1.59 ± 0.71 (1.33, 1.86)	< 0.0001
Lung parenchyma	22.23 ± 5.89 (20.04, 24.43)	38.96 ± 9.09 (35.56, 42.35)	< 0.0001

Data are means ± standard deviation, and the values in parentheses are the 95% confidence intervals. LDCT, low-dose chest CT; IR-HP-CT, iterative reconstruction of high-pitch dual-source chest CT.

* *P-*values were calculated using the Wilcoxon signed-rank test.

### Cardiac pulsation artifacts

**Fig 2:**
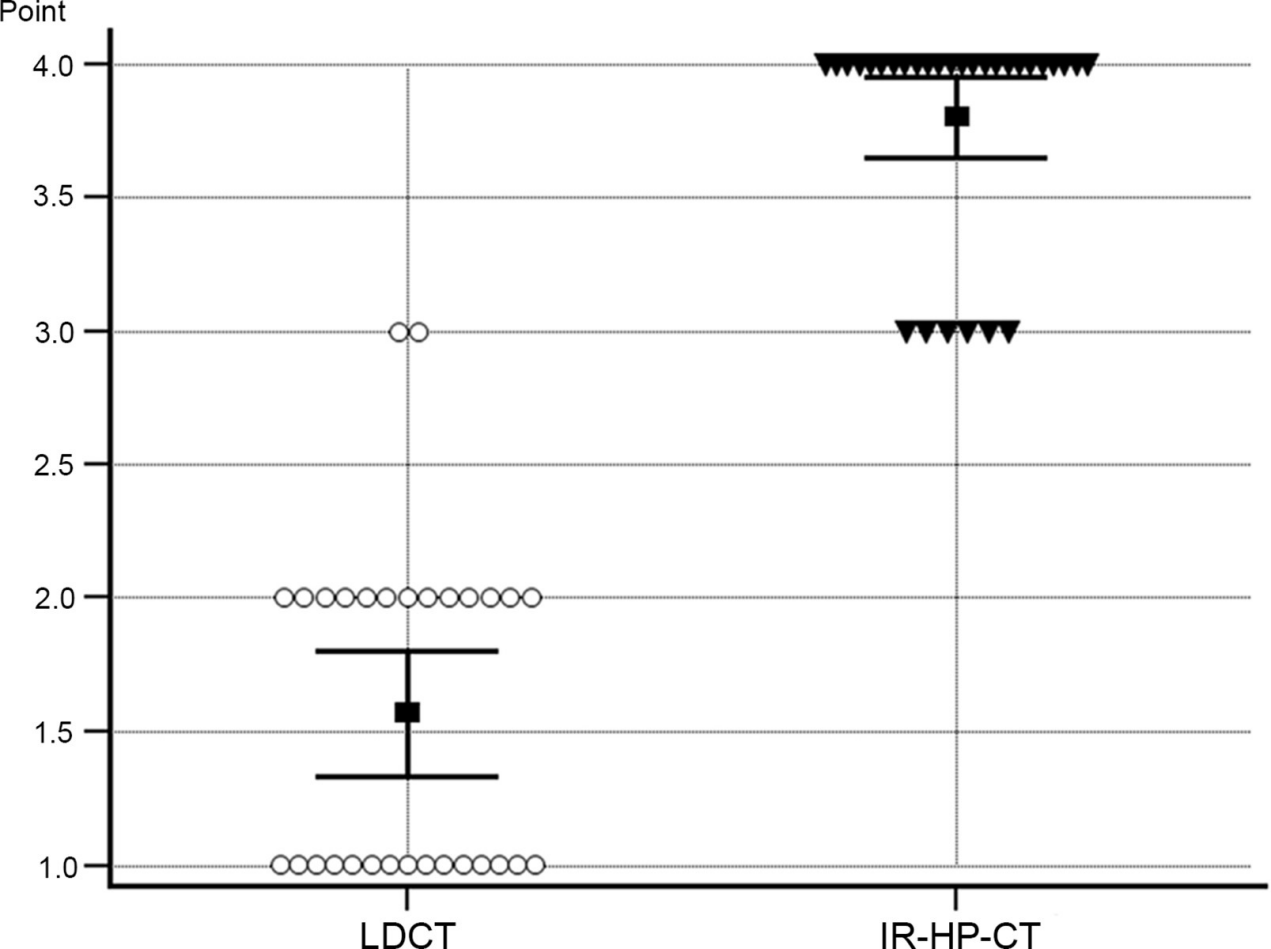
Cardiac pulsation artifacts evident on LDCT and IR-HP-CT scans. IR-HP-CT was associated with a significant reduction in cardiac pulsation (*P* < 0.0001). The black squares are the means and the horizontal bars the 95% confidence intervals.

The score of cardiac pulsation artifacts were significantly reduced on IR-HP-CT (3.8 ± 0.4, 95% CI, 3.7‒4.0) compared with LDCT (1.6 ± 0.6, 95% CI, 1.3‒1.8) (*P* < 0.0001) (Fig 2).

### Subjective image quality

The subjective image quality scores are summarized in Table 5. The overall quality of mediastinum, chest wall, and lung images did not differ significantly between the two scan protocols (*P* > 0.05). The sharpness of mediastinal and chest wall structures and the peripheral lung was significantly higher on IR-HP-CT than LDCT images (*P* < 0.01). IR-HP-CT images contained fewer cardiac pulsation artifacts than those of LDCT images (*P* < 0.01). However, artifacts in the shoulder, chest wall, and lung parenchyma were more common on IR-HP-CT than on LDCT scans (*P* < 0.01). Such artifacts were observed in a few images taken using either protocol; most lung field artifacts were beam-hardening artifacts at the lung apex. In a few IR-HP-CT images, streak artifacts were observed in the basal region of the lung around the diaphragm. The extent of inter-rater agreement in terms of subjective image quality was fair to good for all assessed features (*k* = 0.211 ~ 0.731).

**Table 5:** Subjective image quality scores of LDCT and IR-HP-CT scans.

	LDCT			IR-HP-CT			*P*- value^a^	*K*^b^
	Reader 1	Reader 2	Overall	Reader 1	Reader 2	Overall		
Mediastinum overall image quality	3.07 ± 0.25	3.13 ± 0.35	3.10 ± 0.30	3.07 ± 0.25	3.2 ± 0.41	3.13 ± 0.34	0.527	0.211
Sharp reproduction of mediastinal structures	2.83 ± 0.53	3.07 ± 0.52	2.95 ± 0.53	3.37± 0.41	3.57 ± 0.50	3.47 ± 0.50	0.000	0.626
Chest wall overall image quality	3.07 ± 0.25	3.10 ± 0.31	3.08 ± 0.28	2.90 ± 0.40	3.07 ± 0.37	2.98 ± 0.39	0.058	0.246
Sharp reproduction of chest wall structures	3.17 ± 0.38	3.13 ± 0.35	3.15 ± 0.36	2.90 ± 0.48	3.33 ± 0.55	3.12 ± 0.56	0.637	0.260
Artifacts in the shoulder and chest wall	3.27 ± 0.52	3.20 ± 0.48	3.23 ± 0.50	2.67 ± 0.61	2.83 ± 0.65	2.75 ± 0.63	0.000	0.591
Overall diagnostic image quality of the lung	3.10 ± 0.31	3.33 ± 0.48	3.22 ± 0.42	3.03 ± 0.32	3.47 ± 0.51	3.25 ± 0.47	0.564	0.275
Cardiac pulsation artifacts	3.10 ± 0.31	3.30 ± 0.47	3.20 ± 0.40	3.93 ± 0.25	3.93 ± 0.25	3.93 ± 0.25	0.000	0.731
Artifacts in the lung parenchyma	3.40 ± 0.62	3.30 ± 0.54	3.35 ± 0.58	3.17 ± 0.70	3.07 ± 0.69	3.12 ± 0.69	0.006	0.560
Peripheral lung image sharpness	3.17 ± 0.46	3.13 ± 0.43	3.15 ± 0.44	3.60 ± 0.50	3.63 ± 0.49	3.62 ± 0.49	0.000	0.740

The categories in the first column are those listed in Table 2. Data were presented as means ± standard deviation.

The overall image quality scores are the means of those given by two radiologists. Image quality was evaluated using a four-point scoring system.

LDCT, low-dose chest CT; IR-HP-CT, iterative reconstruction of high-pitch dual-source chest CT.

^a^ *P-*values were calculated using the Wilcoxon signed-rank test.

^b^ Inter-rater agreement (*k*) was evaluated by calculating the *k* statistics.

**Fig 3:**
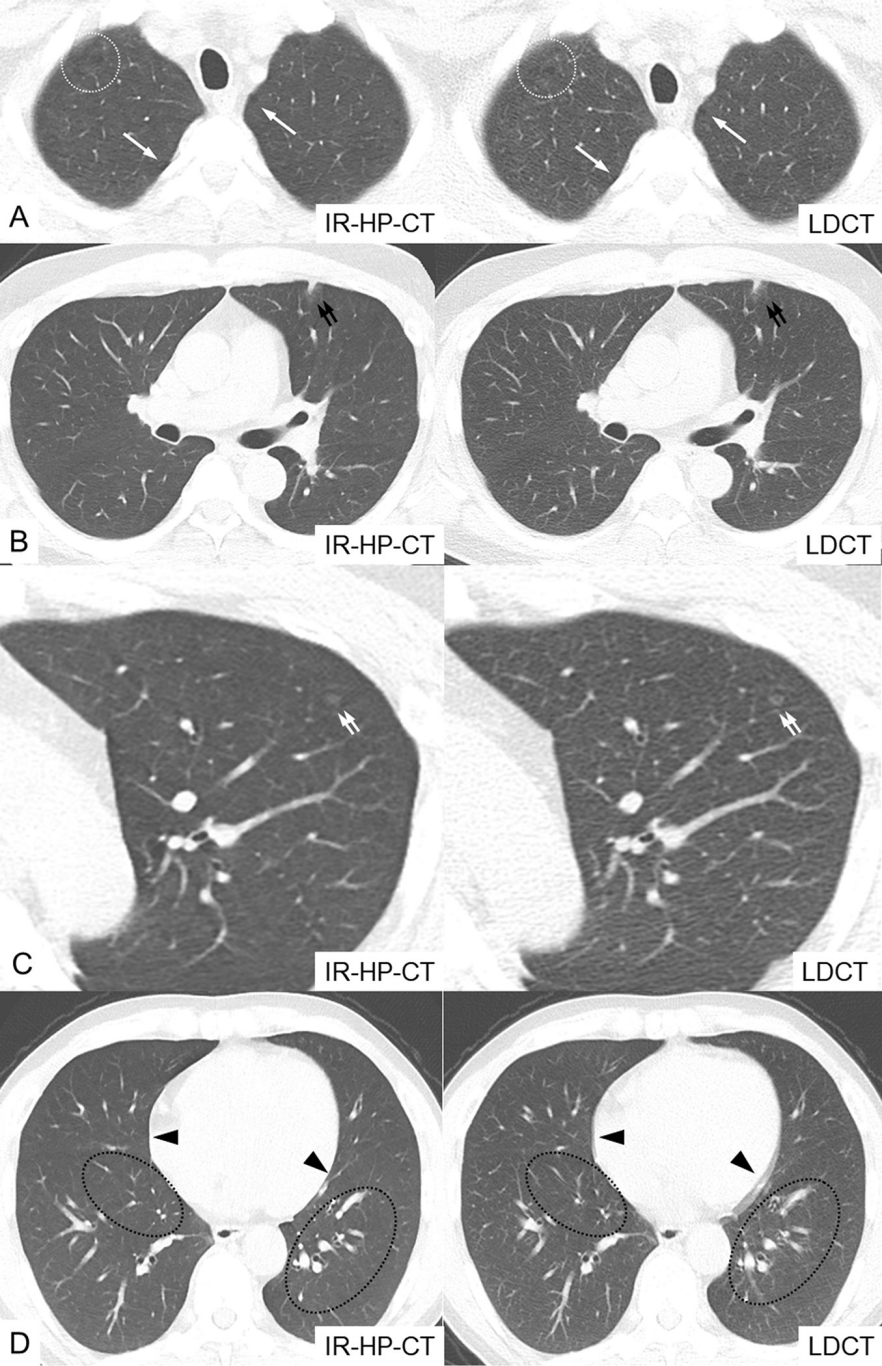
Various pulmonary lesions and cardiac pulsation artifacts detected on IR-HP-CT and LDCT. (A) Small bullae in both upper lobes (white arrows) and centrilobular emphysema in the right upper lobe (dotted circle). (B) Focal nodular consolidation with peripheral ground glass opacity in the subpleural area of the left upper lobe (paired black arrows). (C) A 3-mm-diameter ground glass opacity in the left upper lobe (paired white arrows). (D) IR-HP-CT revealed no cardiac pulsation (4 points), but LDCT was associated with blurring of both cardiac margins (2 points) at the time of cardiac pulsation (black arrowheads). Compared with IR-HP-CT, the LDCT scans exhibited bronchial wall and pulmonary vessel blurring artifacts attributed to cardiac pulsation in the pericardiac areas of both lung fields (dotted ovals).

In the visual assessment of pulmonary lesions, solid nodules, nodules exhibiting ground glass opacity, and ground glass opacity per se were evaluated on a per-lesion basis and emphysema or small bullae on a per-patient basis (Fig 3). The same numbers of solid nodules (n = 15), nodules exhibiting ground glass opacity (n = 2), and ground glass opacities per se (n = 3) were detected in both groups by both radiologists. Small bullae or emphysema were/was evident in fewer subjects on IR-HP-CT (3/30) compared with LDCT (4/30) when the scans were compared by one of the readers. Thus, the effectiveness of pulmonary lesion detection did not differ significantly between the two imaging modalities.

## Discussion

Various techniques are used to reduce the CT radiation dose and improve image quality. Recent studies have focused on reducing the tube voltage or current or on the use of IR algorithms. Voltage and current reductions directly reduce the radiation dose; some preliminary reports on less than 1 mSv LDCT have been available. However, such reductions inevitably increase noise and compromise diagnostic confidence. IR can reduce image noise [26–28].

The DSCT system has been used to reduce the radiation dose to various organs ever since first- and second-generation DSCT systems became available [21, 29–32]. In conditions where kVp and mAs are fixed and IR is applied, pitch and gantry rotation time can affect radiation dose [24]. DSCT has a unique feature; the dose can be reduced by simultaneously increasing the pitch (to 3.2) and decreasing the gantry rotation time (to 0.28 s) compared with LDCT (pitch 0.8 and gantry rotation time 0.5 s). Thus, IR-HP-CT is theoretically superior to LDCT in terms of dose reduction. We previously showed that this was true in practice [23]. When chest CT was performed using high-pitch DSCT, the ED decreased by approximately 13% compared with LDCT employing the same tube voltage and current [23]. In the present study, we reduced the SSDE by approximately 40% by both reducing the effective tube current (from 40 mAs [LDCT] to 30 mAs) and increasing the pitch. Despite the current reduction, the overall image noise and image quality of IR-HP-CT were comparable to or better than those of LDCT, and all pulmonary lesions were effectively detected by both modalities.

In addition, IR-HP-CT performed in the dual-source scan mode at a pitch of 3.2 has the major advantage of not only reducing the radiation dose but also improving temporal resolution; many of the blurring artifacts caused by cardiac pulsation are eliminated [33]. We found that IR-HP-CT improved the sharpness of both mediastinal structures and the lung parenchyma. Although our data are limited, it was not difficult to detect pulmonary parenchymal lesions (the two scan modes did not differ significantly in this context), but one small bulla was detected by IR-HP-CT only. Cardiac pulsation artifacts in lung parenchyma, particularly those near the pulsating free wall of the left ventricle, are not uncommon on chest CT. Such artifacts may simulate bronchiectasis or trigger misinterpretation during pulmonary nodule evaluation [34]. High-pitch scans have previously been reported to reduce the numbers of cardiac pulsation artifacts evident on chest CT of lung parenchyma [23]. This influences the subjective image quality when evaluating the mediastinum and lung parenchyma. On IR-HP-CT, the lung field images were almost motionless and the margins of the mediastinal and peripheral lung parenchyma sharp.

Any reduction in the radiation dose decreases image quality and increases noise. However, this increase can be overcome using IR algorithms [6, 12, 27, 35]. In fact, although the IR-HP-CT tube current was only 30 mAs, IR-HP-CT images were less noisy than LDCT images using a tube current of 40 mAs. This is because the IR-HP-CT images were processed using an IR algorithm and the LDCT images using filtered back projection. IR has been widely used in many areas from children to adults [33]. With combination of various scan-parameter adjustment, IR has two great advantages. First, IR allows radiation dose reduction with preserving imaging quality. Second, IR improves the quality of the images examined under the same scan conditions. IR does not affect the tissue-attenuation value but reduces image noise. Therefore, when IR is applied in the same scan parameters, the CNR, low-contrast resolution and spatial resolution can be improved.[13]. In chest or pediatric CT, IR allows 20–75% reduction of radiation dose compared with filtered back projection [24, 25, 33]. In thorax, CT imaging with IR maintains diagnostic accuracy compared with filtered back projection in the identification and characterization of ground glass opacities, part-solid nodules, and solid nodules, while allowing a dose reduction of approximately 75%. In abdomen, IR has an advantage for lowering noise thus making abdominal CT diagnostically acceptable at reduced radiation dose, especially in arterial phase image [36, 37]. In addition, CT colonography is routinely performed at a reduced dose because of the high contrast between air and colon lumen [38]. IR shows considerable potential in CT pulmonary angiography with estimated radiation savings of 25%–75% and better diagnostic performance [39, 40]. In head and neck, IR allows more than 40% radiation dose reduction, mitigating artifacts, and improving diagnostic performance to detect intracranial hemorrhages [41, 42].

Inevitably, a few instances of artifacts such as beam hardening, streak and quantum mottled artifacts were found around the chest wall and ribs [43]. Although both groups were scanned with the same tube voltage of 120 kVp, IR-HP-CT used fast gantry rotation, relatively lower tube current and high pitch. It is considered that the total radiation energy was reduced by these factors. Relative deficiency of X-ray photon energy and flux induced by the lower tube current and improved temporal resolution may cause of beam hardening, streak and quantum mottled artifacts. However, these artifacts did not interfere with chest CT interpretation or the identification of boundaries between anatomical structures. Thus, these artifacts may not influence the subjective assessment of lung parenchyma image quality when IR-HP-CT is used to evaluate pulmonary lesions.

However, IR-HP-CT performed using the second generation DSCT is associated with a limited scan field. The FOV of the second tube is only 34 cm in length. Thus, the longest distance that can be covered by that tube is 34 cm; tissue outside this area is not reconstructed. In fact, in one of our volunteers, rib cage and chest wall images were lacking. However, structures located within FOV of the second tube has no influence on the geometric measurement. In our study, the SNR and the image noise were measured in the area within 34cm corresponding to FOV of the 2nd tube. Thus, our results were not affected by FOV limitation. This limitation may be overcome using a thick back pad to displace the scan center upwardly, thus moving the largest region of the thoracic cage closer to the center. With the advancement of CT technology, the third generation of DSCT has no limitation of FOV.

This study had several limitations. First, this was a single center study. Thus, external validation will be required. Second, our study had relatively small sample size. However, this study was designed prospectively, and the minimum sample size was determined to be 27 patients based on the one-tailed pre-hoc power analysis using effect size of 0.5, a priory statistical significance of 0.05 and a power of 80% [44]. We enrolled 30 patients with a dropout rate of approximately 10% and obtained 30 pair sets of each IR-HP-CT and LDCT scans. Third, our study population was healthy volunteers so that small number of lung lesions were evaluated. There was no difference in the detecting of pulmonary lesions such as nodules, GGO-nodules, GGO, or emphysema except one bulla was not detected on IR-HP-CT. Thus, the effectiveness of 120 kVp/30 mAs IR-HP-CT in diagnosing pulmonary lesions needs further evaluation.

In conclusion, 120 kVp/30 mAs IR-HP-CT reduced not only the SSDE by 40% but also the cardiac pulsation evident on both lung and mediastinal images, and it afforded an image quality comparable to or superior to that afforded by 120 kVp/40 mAs LDCT.

## Supporting information

S1 File HP-LDCT data. (XLSX)

## Abbreviations

DSCT: dual-source computed tomography
DLP: dose length product
ED: effective radiation dose
FOV: field of view
IR: iterative reconstruction
IR-HP-CT: iterative reconstruction of high-pitch dual-source chest CT
LDCT: low-dose chest CT
SNR: signal-to-noise ratio
SSDE: size-specific dose estimation
